# Application of Nanoparticle Technology to Reduce the Anti-Microbial Resistance through β-Lactam Antibiotic-Polymer Inclusion Nano-Complex

**DOI:** 10.3390/ph11010019

**Published:** 2018-02-10

**Authors:** Constain H. Salamanca, Cristhian J. Yarce, Yony Roman, Andrés F. Davalos, Gustavo R. Rivera

**Affiliations:** 1 Facultad de Ciencias Naturales, Universidad Icesi, Calle 18 No. 122-135, Cali 760031, Colombia; cjyarce@icesi.edu.co (C.J.Y.); yonyroa@yahoo.com (Y.R.); afdavalos@icesi.edu.co (A.F.D.).; 2 SIT Biotech GmbH, BMZ 2 Otto-Hahn-Str. 15, 44227 Dortmund, Germany; gunam04@gmail.com

**Keywords:** polymer-drug association, inclusion nano-complex, an amphiphilic polymer, polysoaps, antibiotic resistance, ampicillin trihydrate

## Abstract

Biocompatible polymeric materials with potential to form functional structures in association with different therapeutic molecules have a high potential for biological, medical and pharmaceutical applications. Therefore, the capability of the inclusion of nano-Complex formed between the sodium salt of poly(maleic acid-*alt*-octadecene) and a β-lactam drug (ampicillin trihydrate) to avoid the chemical and enzymatic degradation and enhance the biological activity were evaluated. PAM-18Na was produced and characterized, as reported previously. The formation of polymeric hydrophobic aggregates in aqueous solution was determined, using pyrene as a fluorescent probe. Furthermore, the formation of polymer-drug nano-complexes was characterized by Differential Scanning Calorimetry-DSC, viscometric, ultrafiltration/centrifugation assays, zeta potential and size measurements were determined by dynamic light scattering-DLS. The PAM-18Na capacity to avoid the chemical degradation was studied through stress stability tests. The enzymatic degradation was evaluated from a pure β-lactamase, while the biological degradation was determined by different β-lactamase producing *Staphylococcus aureus* strains. When ampicillin was associated with PAM-18Na, the half-life time in acidic conditions increased, whereas both the enzymatic degradation and the minimum inhibitory concentration decreased to a 90 and 75%, respectively. These results suggest a promissory capability of this polymer to protect the β-lactam drugs against chemical, enzymatic and biological degradation.

## 1. Introduction

Nowadays, bacterial antibiotic resistance stands as a significant public health problem in our society and, the considerable challenge of finding new antibiotic molecules or improving the activity of the existing ones demands a joint effort of multiple disciplines [1,2,3]. Antibiotic resistance results naturally from the inherent ability of bacteria to multiply rapidly and mutate as an adaptation strategy. However, patient misuse, the doctors' mis-prescription and their overuse in the food industry have exacerbated the problem of antibiotic resistance [2]. This issue is becoming even more significant due to the recent decrease in research efforts to produce new antibiotics [4]. Pharmaceutical companies, government agencies and academia are not investing enough resources to face the emerging strains of multidrug-resistant “superbugs”. Many bacteria show resistance to antibiotics but *Staphylococcus aureus* stands as one of the most relevant due to the high morbidity and mortality due to its bacteremia [5,6]. The high prevalence of *S. aureus* in nosocomial infections and its high rate of penicillin resistance makes this pathogen the primary cause of resistant bacteria-related diseases, worldwide. The mechanisms of bacterial resistance are multiple and can be conjugate but the most common one involves the production of β-lactamases. These are a series of enzymes, which can hydrolyze the β-lactam ring present in the penicillin-like antibiotics [7]. 

Several alternatives have been developed to treat infections with antibiotics resistant to *S. aureus*. Initially, the first line of defense was the use of β-lactamase resistant molecules like methicillin and oxacillin. However, only two years after their introduction a *S. aureus* strain resistant to methicillin (MRSA) emerged [8,9,10]. Today, MRSA strains are endemic in hospitals worldwide. MRSA infections are commonly treated with non-β-lactam antibiotics like clindamycin but there are resistant strains as well. These strategies involve the use of new molecules that merely test the refined ability of the microbes to evolve and adapt. The continuous emergence of resistant strains highlights the need for the development of new strategies to treat bacterial infections. Recent plans that seek to improve the effectiveness of conventional antibiotics against resistant bacteria include their use along with inhibitors of β-lactamases [11,12] and, to a lesser extent, the control of β-lactamase expression [13,14]. However, there is few available information on the use of polymeric materials to avoid antibiotic biological degradation [15,16,17]. In aqueous solution, some amphiphilic polymers like polysoaps, hydrophobically modified polymers and block polymers may form hydrophobic pseudo-phases capable of solubilizing organic molecules [18,19,20,21,22,23,24].

The sodium salt of poly(maleic acid-*alt*-octadecene), named here as PAM-18Na, can form different hydrophobic “pseudo-phases” in a concentration-dependent way. At very low concentrations, PAM-18Na forms unimolecular aggregates, i.e. each polymer chain collapses forming a compact coil. Whereas, in more concentrated solutions, PAM-18Na forms multimolecular aggregates. This polymer has also been able to solubilize different organic molecules such as alkyl-phenols [25] and *N*-alkyl-nitroimidazoles [22]. Based on these observations we hypothesized that PAM-18Na polymer could be useful to protect β-lactam antibiotics from the action of β-lactamases and thus improves their effect on antibiotic-resistant bacteria. In this work, we evaluated the ability of the PAM-18Na polymer to prevent the chemical and enzymatic degradation of ampicillin trihydrate when subjected to severe acid conditions and purified β-lactamase obtained from *P. fluorescens*, respectively. Likewise, we compared the effect of the PAM-18Na polymer on the antibiotic activity of AT over several *S. aureus* strains. Our results suggest a promissory capability the polymer PAM-18Na to protect the β-lactam drugs against chemical and biological degradation through the formation of a polymer-drug inclusion nano-complex in aqueous media.

## 2. Materials and Methods

### 2.1. Materials

Poly(maleic anhydride-*alt*-octadecene) denominated like PAM-18 with average Mw 30,000–50,000 and Lucifer yellow were obtained from Sigma-Aldrich^®^, ampicillin trihydrate—here referred to as AT—was from Fersinsa Gb^®^ (Coahuila, Mexico), recombinant β-lactamase from *Pseudomonas fluorescens* was obtained from Sigma^®^ (Saint Louis, MO, USA). It was received lyophilized and suspended according to manufacturer indications. Ultrapure water was obtained with an Elix Essential Millipore^®^ (Darmstadt, Germany) purification system. All other reagents were from Merck^®^ (Kenilworth, NJ, USA). *Bacterial strains: S. aureus* strains ATCC 25923, ATCC 29213 y ATCC 43300 were purchased from Microbiologics Inc.^©^ (St Cloud, MN, USA) and were reconstituted according to the instructions. 

### 2.2. Obtention and FTIR Characterization of PAM-18Na Polymer

PAM-18Na was obtained as previously described [22]. Briefly, 100 g of PAM-18 was hydrolyzed in 2 L of ultrapure water mixed with NaOH in a 1:1 molar ratio (according to PAM-18 copolymer unit), where the polymeric material obtained was named PAM-18Na. The modification was carried out at room temperature for 24 h under moderate agitation (200 rpm). Subsequently, the polymer solution was dialyzed using cellulose membrane (12 kD cut off size) and pre-concentrated through a stirred ultrafiltration cell (Amicon^®^ cells 8400, Merk-Millipore, Billerica, MA, USA) with a 12-kDa cut-off polyethersulfone (PES) membrane. Subsequently, the polymer solution was lyophilized (model FDU 1110, Eyela, Tokyo Rikakikai, Tokyo, Japan) until obtaining solid materials with a yield greater than 90%, which was sieved with 75 μm mesh (number 200).

### 2.3. Preparation of Inclusion Nano-Complexes in Aqueous Media

The inclusion nano-complexes between TA and the PAM-18Na polymer were formed in situ. For this, a defined amount of PAM-18Na polymer was added in ultra-pure water until reaching a homogeneous dispersion with desired concentration. Then, the β-lactam drug was added “little by little” to the polymeric dispersion, using moderate magnetic stirring (200 rpm) at room temperature until obtaining a translucent dispersion.

### 2.4. Steady-State Fluorescence Assay

The presence of polymeric hydrophobic aggregates in aqueous media formed by the PAM-18Na was evidenced through by the steady-state fluorescence study using a microplate reader (Synergy h1 hybrid multi-mode) and pyrene as a fluorescent probe. A stock solution of pyrene (2.66 × 10^−5^ M) was prepared to which micro-volumes of PAM-18Na polymeric solution (1 mg/mL) were added until a pyrene concentration of 1.33 × 10^−6^ M was obtained. The excitation wavelength was set at 337 nm and the intensities of the third (I_3_) and first (I_1_) peaks of the pyrene emission spectrum, (at 382 nm and 373 nm, respectively) were measured.

### 2.5. Characterization of Drug-Polymer Inclusion Complex

#### 2.5.1. Thermal Characterization of the Polymer-Drug Blend

PAM-18Na polymer, AT and polymer-drug solid mixture in different proportions was studied on a DSC Q2000 (TA Instruments) calibrated with indium T_m_ = 155.78 °C, ΔH_m_ = 28.71 J/g. The DSC analysis was performed using three cycles of heating and cooling from −90 °C (183.15 ° K) to 200 °C (523.15 ° K) with a heating rate of 20 °C/min.

#### 2.5.2. Association Efficiency

Independent solutions of AT and PAM-18Na polymer were prepared using several phosphate buffer solutions, having pH values of 4.0, 7.0 and 10.0. Each solution was fixed to an ionic strength of 10 mM. For AT, the solution concentration was 40 µg/mL, while the PAM-18Na amount was set to form a 1:1 polymer-drug molar ratio according to PAM-18Na co-monomeric unit. Equal volumes of both solutions were mixed by ultrasonic stirring for 1 h. Then, each solution was settled inside an ultrafiltration tube (VWR, Modified PES 10 kDa, 500 µL) and centrifuged at 9000 G (10.000 rpm) for 7 min. From the filtrate obtained (lower fraction in the ultrafiltration tube), to quantify the amount of AT. The absorbance was measured in a microplate reader (Synergy h1 hybrid multi-mode) at a wavelength of 256 nm and the amount of AT was determined by interpolation from the calibration curve built at concentrations of 2, 5, 10, 20 y 40 μg/mL. The amount of AT encapsulated or contained in the PAM-18Na polymeric hydrophobic aggregates was calculated using the following expression:

(1) $$AE=\left[\frac{\left(Q_{t}-Q_{s}\right)}{Q_{t}}\right]\times 100\%$$

where *AE* corresponds to the association efficiency, *Q_t_* is the initial total amount of added *AT* and *Q_s_* is the filtrated amount after centrifugation.

#### 2.5.3. Zeta Potential and Size Measurements

Each of the zeta measures was performed in triplicate using a Zetasizer Nano ZSP (Malvern Instrument, Malvern, UK) at 25 °C. The first part of the study was focused on characterizing PAM-18Na polymer in aqueous media regarding the pH of the media. In this case, 40 µg/mL polymer solutions were prepared, where the pH was adjusted with concentrated solutions of NaOH and HCl and slightly shaken for 48 h. The second study was focused on the characterization of the drug-polymer interactions between PAM18-Na and ampicillin trihydrate in aqueous media. In this case, each solution was prepared using several phosphate buffer solutions, with different pH values of 4.0, 7.0 and 10.0 and was fixed to the ionic strength of 10 mM. Each measurement was performed with freshly prepared samples. To the aim of evaluating the impact of AT loading into the hydrophobic polymeric aggregates formed by PAM-18Na, different amounts of AT were added until achieving a final concentration of 0.23 µg/mL, corresponding to 1:1 polymer-drug molar ratio according to the PAM-18Na co-monomeric unit. Each of these studies was carried out by auto-titration with independent cells, where measurements of size were carried out using a quartz flow cell (ZEN0023), while the zeta potential was carried out with a disposable folded capillary cell (DTS1070). 

#### 2.5.4. Viscometric Measurements

The characterization of the polymer-drug interactions was also studied by viscosimetry. A viscometer (microVisc TC, RheoSense, San Francisco, CA, USA) coupled to a low viscosity chip (16HA05100243) was used. Each measurement was performed using a 1:1 polymer-drug molar ratio according to a co-monomeric unit of PAM-18Na at 25 °C and at different pH values (1, 4, 7 and 10), with freshly prepared samples and in triplicate as described above.

### 2.6. Degradation Assays

#### 2.6.1. Chemical Degradation Assay

Due to the remarkable degradability of the beta-lactam ring in AT respect to temperature and media pH [26], a stability test to stress conditions, in the presence and absence of the PAM-18Na polymer was performed to evaluate the potential of this polymer as protector of the β-lactam drugs. For this, different initial concentrations of AT (1, 3 and 5 mg/mL) in a strong acidic media (pH 1.2) were prepared and stirred for 6 h at 40 °C. Consecutively, each sample was taken every 10 min and analyzed by UHPLC with a photo-diode-array detector (Lachrom ultra Hitachi, VWR, Tokio, Japon). This assay was performed by in triplicate.

#### 2.6.2. Enzymatic Degradation Assay

The β-Lactamase activity was monitored by measuring the hydrolysis of the β–lactam ring of ampicillin at 204 nm, as reported previously [27,28]. The assays were carried out by mixing 125 µL of a β-Lactamase solution (38 µg/mL) with a solution containing 800 µL of AT (81.25 µM) and 1325 µL of phosphate buffer 50 mM (pH 7.0) in a quartz cell. The reactions were carried out at 25 °C for 20 min, measuring the absorbance every 2 min using a UV spectrophotometry (Shimadzu UV model 1800) coupled to a temperature control system). Initial rates (*v*_0_) were calculated using the linear portion of the absorbance vs. time plot for each enzymatic reaction. The β-Lactamase activity units (U) were defined as the amount of enzyme that hydrolyzes 1.0 nmol of AT per minute at 25 °C and pH 7.0. The AT concentration in the enzymatic assay was calculated using a standard curve made by measuring the absorbance at 204 nm of standard solutions ranging from 8.13 to 81.25 µM. The β-Lactamase activity in the presence of the PAM-18Na polymer was calculated as described above but using a solution prepared by mixing ampicillin and the polymer in a 1:1 molar ratio in phosphate buffer 50 mM (pH 7.0). 

#### 2.6.3. Biological Degradation Assays

##### Ampicillin Susceptibility

AT susceptibility for each of the *S. aureus* strains used in this study (*S. aureus* ATCC 25923, *S. aureus ATCC* 29213 and *S. aureus* ATCC 43300) was measured following the guidelines of the Clinical and Laboratory Standards Institute (CLSI) [29]. In brief, a culture was grown in a petri dish using Mueller-Hinton (Scharlab^®^, Barcelona, Spain) agar and the diameter of the inhibition halo around an ampicillin Sensi-Disc (BD) was measured. For each bacterial strain, the average of four replicates was used in the analysis. 

##### β-Lactamase Production

The β-Lactamase production, in each of the four *S. aureus* strains, was assayed by the chromogenic cephalosporin nitrocefin [30,31]. For each strain, three colonies were inoculated on top of a Nitrocefin disc (Abtek Biologicals, Liverpool, UK) and a color change was monitored as an indicator of β-Lactamase enzymatic activity according to manufacturer instructions.

##### Minimum Inhibitory Concentration (MIC)

Ampicillin trihydrate MIC was determined by the broth microdilution method according to the CLSI guidelines [32]. In each case, the MIC was determined for AT, PAM-18Na polymer and the mix of PAM-18Na and AT, in a 1:1 ratio (based on the copolymer unit), as previously described. The concentrations evaluated were 0.0625, 0.125, 0.25, 0.35, 0.5, 2, 8, 32, 128, 192, 256 µg/mL, respectively. The assays were performed in 96 round bottom well plates (BD) using 500 µL of the appropriate *S. aureus* strain grown in Mueller-Hinton broth (Scharlau^®^) at a turbidity of 0.1 absorbance units. Twenty-four replicates were performed for each concentration and visually inspected for the presence of a bacterial cell pellet. 

### 2.7. Data Analysis

Data analysis for MIC was carried out using the Microsoft^®^ Excel and Statgraphics Centurion XV (Version 15.2.06 software). The effect of ampicillin, PAM-18Na polymer and polymer-drug mix, on culture growth, was determined with a 95% confidence interval.

## 3. Results and Discussion

### 3.1. Obtention and FTIR Characterization of PAM-18Na Polymer

Formation of PAM-18Na polymer was evidenced by a physical change. The solution passes from a heterogeneous mixture to an utterly homogenous solution with a yellowish color, due to the opening of the maleic group in PAM-18 which produces carboxylic acid and carboxylate groups in the polymer backbone. This structural modification was inferred by comparing the FTIR spectra of both PAM-18 and PAM-18Na polymers according to previous reports (Supplementary Materials). The results showed the typical symmetric and asymmetric stretching values characteristic of the hydro-carbonated alkyl chain at 2920 and 2848 cm^−1^. Upon the reaction, two new signals of the maleic anhydride carbonyl groups at 1773 and 1704 cm^−1^ to 1706 and 1556 cm^−1^ supports the formation of the carboxylic acid and carboxylate species. Likewise, the typical broad signal of the hydroxyl group of carboxylic acid at 3110 cm^−1^ is observed, corroborating that PAM-18Na polymer presents both the carboxylic acid and carboxylate form.

### 3.2. Steady-State Fluorescence Assay

**Figure 1:**
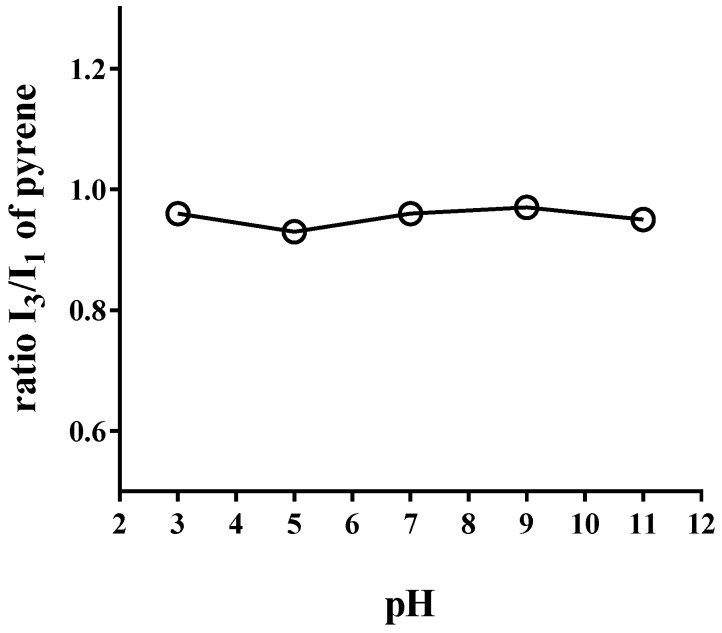
I_3_/I_1_ pyrene ratio of emission spectra in aqueous solutions of PAM-18Na at different pH and solvent with different polarity degree.

The I_3_/I_1_ pyrene ratio of emission spectra is strongly dependent on the medium polarity and it has been used to establish an empirical polarity scale that is widely used in the study of microheterogeneous systems [33]. The I_3_/I_1_ pyrene ratio in aqueous solutions of PAM-18Na at different pH is shown in Figure 1. For the sake of comparison, the spectra were normalized using the intensity of the peak located at 373 nm. The results show that PAM-18Na might create aggregates in aqueous media with a similar polarity like that described by pyrene in butanol (~0.95). However, this effect is trivial to those previously found in a similar polymer [34]. On the other hand, the I_3_/I_1_ ratio does not show a considerable change concerning the media pH, suggesting that the hydrophobicity of polymeric aggregates tends to remain constant independently of the pH. Hence is possible to solubilize or contain small organic molecules.

### 3.3. Characterization of the Drug-Polymer Inclusion Complex

#### 3.3.1. Thermal Characterization of the Polymer-Drug Blend

**Figure 2:**
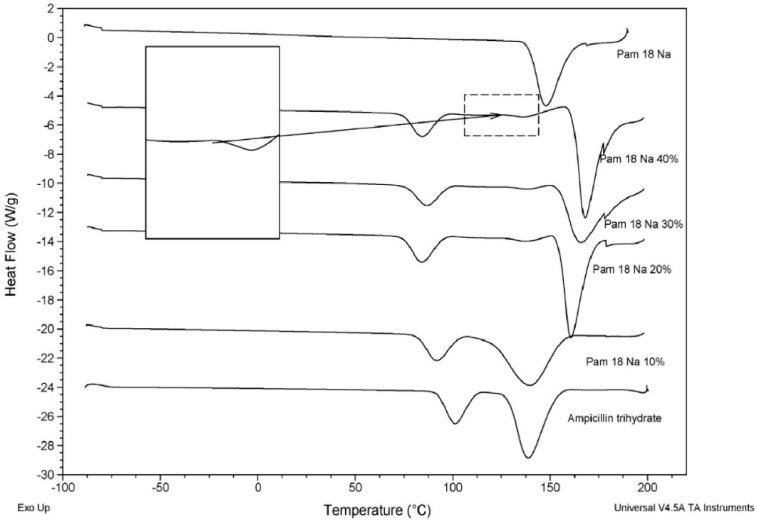
DSC Thermograms of ampicillin trihydrate and PAM-18Na polymer in solid blends at different proportions.

The analysis of thermal characterization for ampicillin trihydrate in different proportions of PAM-18Na polymer is shown in Figure 2.

From the DCS thermograms, it is possible to observe two phenomena: (i) a decrease in the thermal transition temperature of the PAM-18Na polymer around 175 °C and a higher thermal transition temperature of AT around 137.5 °C, which becomes stronger with the increase of the polymer amount; (ii) The appearance of a new thermal signal that increases its intensity and energy with the rise in the polymer amount. These results suggest an intense interaction between the PAM-18Na polymer and AT. 

#### 3.3.2. Encapsulation Efficiency

**Figure 3:**
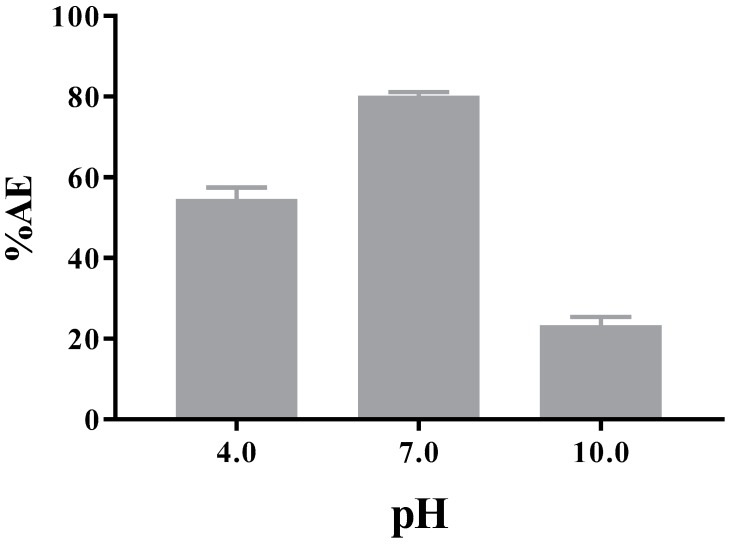
Association efficiency of ampicillin trihydrate by the PAM-18Na polymer in aqueous media to different pH values at 25 °C.

The results of ampicillin trihydrate associated or contained into the PAM-18Na polymeric hydrophobic aggregates are shown in Figure 3, where the results shown a dependence of the AT association efficiency regarding media pH, being higher at pH 7.0 than pH 4.0 and 12.0, respectively. The above mentioned is a fascinating result because the PAM-18Na polymer has shown the capacity to form hydrophobic aggregates or “hydrophobic domains” independent of pH. Therefore, a high association degree should be expected, as has been observed in similar studies [21,22]. On the other hand, these differences can be explained according to (i) the amphoteric nature of ampicillin and (ii) the type of polymer-drug complex formed at each pH. In the first case, the ability of AT to transfer from aqueous phase to the polymeric hydrophobic pseudo-phase will depend on its ionization degree, where at pH close to 7.0, the AT tends a neutral form, favoring its incorporation into the PAM-18Na polymeric pseudo-phase. Based on the measured value of ~0.95 in the I_3_/I_1_ pyrene ratio of, the polarity of the pseudo-phases presents a favorable environment to incorporate organic molecules like ampicillin. Therefore, our results also suggest that hydrophobic domains are formed by the PAM-18Na might associate or contain a significant amount of AT. 

Also, it was possible to appreciate a very interesting situation related to the organoleptic characteristics of AT, which has a typical strong smell. This effect was a considerable loss of such characteristic odor in the presence of polymer PAM-18Na, disappeared almost entirely at pH: 7.4. This result suggests indirectly, the formation of a complex between the PAM-18Na polymer and the drug. Regarding the type of polymer-drug complex formed at each pH, it is necessary to consider the effects of size, zeta potential and viscosity of the PAM-18Na system with and without drug at different pH, which is explained as follows. Finally, we must comment that the evaluations at pH values of 1.2 and 12 were not possible because the integrity of the ultrafiltration membrane was affected by those pH values.

#### 3.3.3. Zeta Potential and Size Measurements

**Figure 4:**
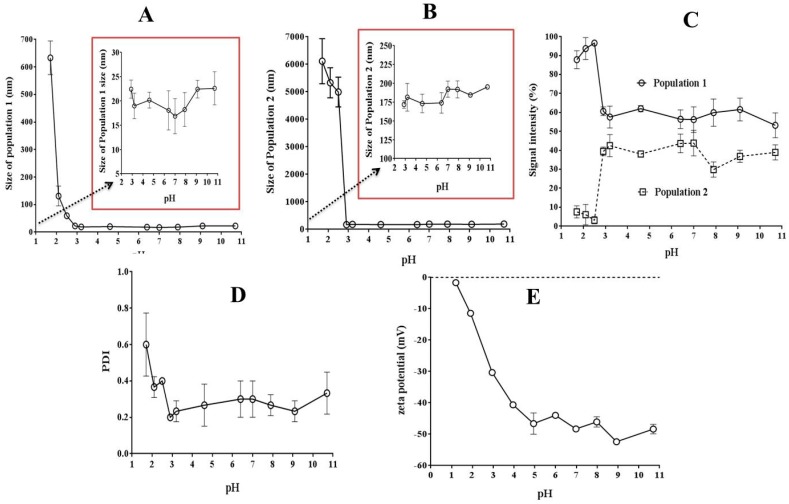
Size, signal intensity, polydispersity (PDI) and zeta potential of PAM-18Na in aqueous media regarding media pH at 25 °C.

It is imperative to highlight that it is common to assume that all association processes between organic substrates and polymers, such as PAM18Na, occur through solubilization within the polymer hydrophobic domain formed in aqueous media. However, there is another possibility of an association—such as the adsorption on the polymer-solvent interface—as we have already demonstrated for ampicillin with a cationic polymer, such as Eudragit E, where the drug-polymer association is mainly given on this interface [35]. The first study was focused on evaluating the effect of pH on the size and zeta potential of PAM-18Na polymer in solution with the aim of elucidating the association mechanism. These results are shown in Figure 4. In the case of size measurements, a bimodal size distribution was observed, in the range of pH between 3 and ~11. Here, the first population was around 20 nm (Figure 4A), while the second population was approximately 200 nm (Figure 4B). When there was pH below ~3, an increase of both size populations was observed, the first one went from ~20 nm to ~600 nm and the second from ~200 to ~6000 nm. Also, the signal intensity of size and polydispersity (Figure 4C,D) in both populations were strongly affected by extreme acidic conditions. In the case of the first population, an increase in signal intensity from ~60% to almost ~95% occurs; here the particles ranging from ~20 nm to ~200 nm possibly corresponds to individual polymer coiled chains that begin to several polymer chains. This result is consistent with the fluorescence studies, using the pyrene probe, where a hydrophobic environment at such pH was observed. In the case second population, a decrease in the signal intensity of size from ~40% to almost ~5% occurs, suggesting the formation of a heterogeneous system like “coarse colloid,” with particles ranging from ~200 nm to ~6000 nm. According to the zeta potential measurements (Figure 4E), the expected behavior was observed because PAM18Na is a polyanion the charges of which can be neutralized due to high proton concentration, as reflected in the values of zeta potential. 

**Figure 5:**
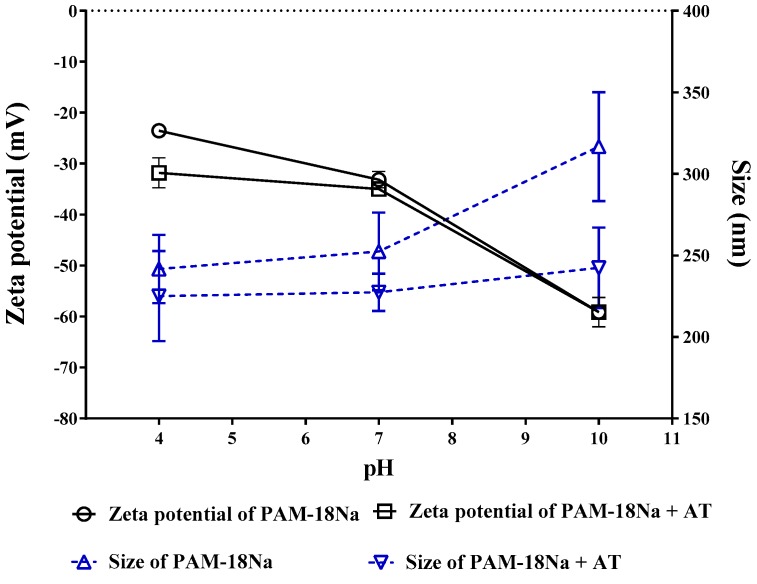
Influence of pH on size and zeta potential values of PAM-18Na in aqueous media in absence and presence of 1:1 molar ratio of Ampicillin Trihydrate at 25 °C.

On the other hand, the second study was a focus on the characterization of the drug-polymer complex between PAM18-Na and AT in aqueous media at 25 °C and different pH values. The results are shown in Figure 5. The results showed that addition of stoichiometric amounts of AT to the PAM-18Na does not affect either the size or the zeta potential in solution. In fact, for the second population, there is a slight change from ~250nm to ~320 nm. Since no relevant differences on the surface properties of the nanoparticles were observed, it is possible to indicate that the association mechanisms between PAM-18Na and AT were driven by the incorporation of the drug within the hydrophobic core of the nano-aggregate formed by the polymer. This result is a similar phenomenon as solubilization in an inclusion nano-complex.

#### 3.3.4. Viscometric Measurements

**Figure 6:**
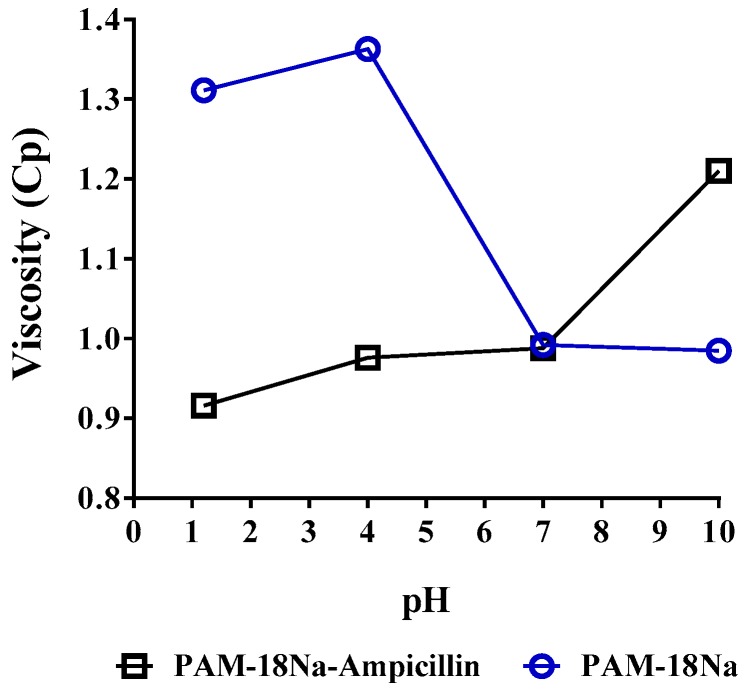
Viscometric Profile of PAM-18 aqueous at different pH in the absence and presence of Ampicillin trihydrate in 1:1 molar ratio at 25 °C.

The viscometric study of PAM-18Na polymeric solutions in the absence and presence of ampicillin trihydrate at different pH values are shown in Figure 6. In the case of PAM-18Na polymer alone in aqueous media with pH values from 1 to 4, the viscosities varied between 1.31 and 1.41 Cp, whereas at pH values from 4 to 7, viscosity decreased to 0.95 at which point it remained constant. Under acidic conditions, PAM-18Na is practically neutral (zeta potential ~0) forming a heterogeneous system like a coarse colloid, where aggregation is highly favored thermodynamically and thus affecting the fluidity of the dispersion media. Otherwise, at pH values from 4 to 7, the PAM-18Na polymer begins to change from a coarse colloid to a nano-dispersion and at pH above 7, the system turns into the polymer solution, where the aqueous media might flow more smoothly. In the case of the polymer-drug mixture at pH values from 1 to 4, the viscosity is lower than that shown by the PAM-18Na polymer alone. This effect could be due to the AT entails a more structured organization in the system avoiding aggregation among particles in the coarse colloid. In the case of pH values above 7, the system becomes a polymeric solution, where AT might be solubilized into the hydrophobic or adsorbed to the polymer/solvent interface, acting as a cross-linker forming a gel and then increasing the viscosity. These results are consistent with the slight increase in size observed in the second population of particles.

### 3.4. Degradation Assays

#### 3.4.1. Chemical Stability

**Figure 7:**
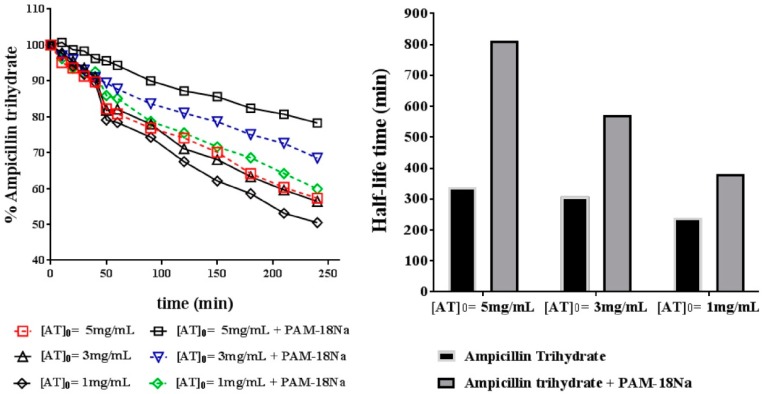
(**Left**) degradation profile of ampicillin trihydrate (AT) at different concentrations under acidic conditions, pH: 1.2 at 40 °C. (**Right**) calculated half-life of AT under same conditions.

The chemical degradation profile of AT in presence and absence of PAM-18Na polymer, using several initial concentrations of ampicillin to stress conditions are shown in Figure 7. The kinetic analysis of the degradation profiles was analyzed by using an integral method, where each model was fixed to a second-order equation. 

The results show that the half-life increases in the presence of PAM-18Na polymer. It is also noted that, as the initial amount of the drug increased, the average half-life time increased, which is a very interesting, because it has been reported that this drug accelerates the hydrolytic degradation concerning its concentration [26]. Therefore, these results suggest that degradation of this drug that is highly susceptible to the acid hydrolysis is avoided by its incorporation into PAM-18Na polymeric nano-aggregates.

#### 3.4.2. β-Lactamase Activity Assay

**Figure 8:**
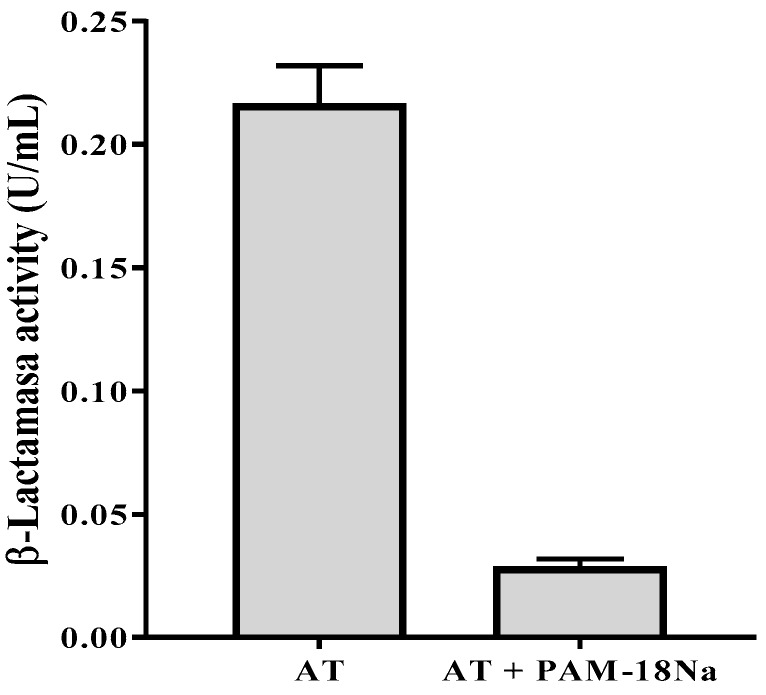
Effect of the polymer PAM-18Na on the activity of the *P. fluorescens* β-lactamase.

Based on the previous results suggesting the formation of an inclusion nano-complex between PAM-18Na polymer and AT, the capability of this polymer to protect the antibiotic against the enzymatic activity of a β-Lactamase was tested. The ability of the class C β-Lactamase (obtained from *P. fluorescens)* to hydrolyze ampicillin has been well established [28]. In our study, the β-Lactamase from *P. fluorescens* showed an enzymatic activity of 0.207 ± 0.039 U/mL over the substrate (ampicillin trihydrate). Remarkably, the enzymatic activity significantly decreased to 0.029 ± 0.001 U/mL when the AT was incorporated into the polymer PAM-18Na. This result represents an 86% decrease in the capacity of the β-Lactamase to hydrolyze AT once is encapsulated in PAM-18Na (Figure 8). 

#### 3.4.3. Antimicrobial Activity Assays

##### Strains Characterization

The *S. aureus* strains used in this study were classified as ampicillin resistant or susceptible according to the parameters reported in the M100 guide from the CLSI [36]. According to this guide, a strain is classified as resistant when the diameter of the halo around a 10-µg ampicillin Sensi-Disc is ≤28 mm, or sensitive when this width is ≥29 mm. Based on the results reported in Table 1, *S. aureus* strain ATCC 25923 is susceptible to ampicillin. Whereas, *S. aureus* strains ATCC 29213 and ATCC 43300 are resistant to the antibiotic.

**Figure 9:**
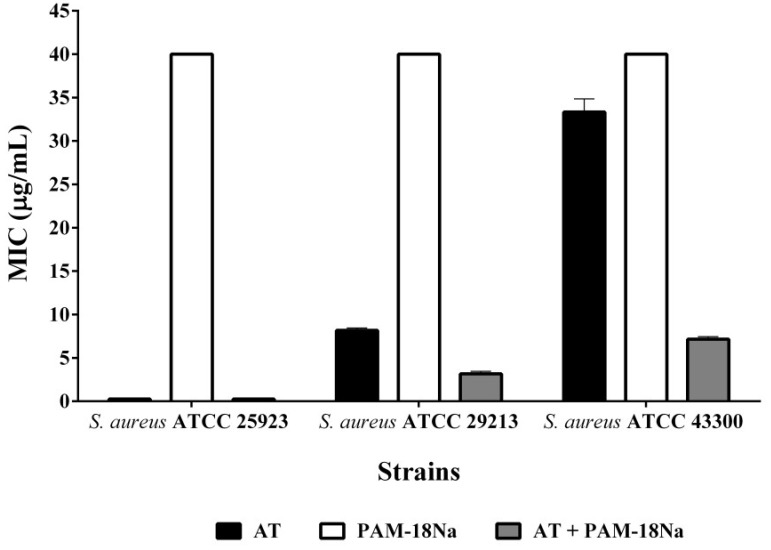
Minimum Inhibitory Concentration (MIC) of ampicillin trihydrate (AT) in the absence and presence of the PAM-18Na polymer in different *S. aureus* Strains.

We evaluated the effect of the polymer PAM-18Na on the ampicillin MIC for the three *S. aureus* strains described here. In the case of the sensitive strain, the presence of the polymer did not affect the ampicillin MIC. On the other hand, when PAM-18Na was used in a 1:1 ratio with ampicillin the MIC decreased to 75% for both resistant strains (Figure 9). In all cases, PAM-18Na by itself showed no antibiotic effect up to the maximum concentration evaluated (256 µg/mL).

The ampicillin resistance in the strains evaluated arises mainly from β-Lactamase secretion. Thus, the improvement in antibiotic activity observed for the combination of PAM-18Na and ampicillin can be explained by the capacity of the polymer to interact with the antibiotic and protect it from enzymatic cleavage. Complementary, the ATCC 43300 strain showed a higher ampicillin-PAM-18Na tolerance than that of ATCC 29213. This observation might be a consequence of the second resistance mechanism present in the former strain, which involves penicillin-binding protein modification. In the resistant strains tested the β-Lactamase is secreted by the media. Therefore, PAM-18Na protection is most likely taking place outside the microorganism. Future studies are undertaken to a better understanding of the way the polymer delivers its cargo to complete the picture of pharmacological activity for the PAM-18Na-ampicillin association.

Altogether, the results presented here support the association of the polymer PAM-18Na and ampicillin in solution. Also, they suggest that this polymer can be useful to improve the antibiotic activity of traditional drugs against their resistant bacteria. Similar results, with chemically related polymers, have been reported previously. For example, β–lactam antibiotics in association with polyacrylate nanoparticles [37,38] or vehiculated in liposomes [39,40] showed increased effectiveness against methicillin-resistant *S. aureus* strains. Likewise, ampicillin associated with amphiphilic polymers, made by maleic acid and 2-vinylpyrrolidone, showed a marked increment of the antimicrobial activity of ampicillin on clinical isolates of resistant *S. aureus* [16]. Overall, these results highlight the ability of amphiphilic polymers, like PAM-18Na, to increase the antibiotic effect of ampicillin on resistant bacterial strains. 

On the other hand, the high MIC values for PAM-18Na polymer alone, indicate that it has no antimicrobial properties and possibly is a biocompatible material since its chemical characteristics are very similar to others [41,42]. However, this is an effect that will need to be assessed given its potential. Finally, due to the current problem of antimicrobial resistance to antibiotic drugs, the polymer PAM-18Na might be an exciting alternative to improve this effect. Also, it might be a very interesting alternative for those antibiotic β-lactams that are becoming obsolete, where their activity can be enhanced with the development of “smart pharmaceutical formulations.” 

## 4. Conclusions

Here we demonstrated the formation of hydrophobic nano-aggregates in aqueous media by PAM-18Na, which could generate nanoparticles as polymeric intra-aggregates that efficiently incorporated ampicillin trihydrate, leading to the loss of organoleptic characteristics, as well as the avoidance of its degradation by: (i) extreme acidic conditions and (ii) several enzyme beta-lactamases. Furthermore, the complex formed has a size of around 200 nm and a slightly negative neutral surface charge. Finally, our results suggest that the polymer PAM-18Na can be a useful alternative to increase the effectiveness of conventional antibiotics against resistant bacterial strains.

## Figures and Tables

**Table 1:** Ampicillin disk diffusion tests for various *S. aureus* strains.

Strain	Average Diameter (mm)	Standard Deviation
*S. aureus* ATCC 25923 (sensitive)	33.99	0.52
*S. aureus* ATCC 29213 (resistant)	20.15	0.93
*S. aureus* ATCC 43300 (resistant)	12.19	0.51

## Supplementary Materials

The following is available online at http://www.mdpi.com/1424-8247/11/1/19/s1
