# Supplementary material for: Application of Nanoparticle Technology to Reduce the Anti-Microbial Resistance through β-Lactam Antibiotic-Polymer Inclusion Nano-Complex
Source: Pharmaceuticals (Basel). 2018 Feb 10;11(1):19. doi: 10.3390/ph11010019 (PMC5874715; doi:10.3390/ph11010019)
Supplement: Supplementary file 1 [file pharmaceuticals-11-00019-s001.pdf]

Protective ability over chemical, enzymatic and biological degradation of a model β-lactam antibiotic by inclusion nano-complex

Supplement material.

1. Support information for IR spectrum of PAM-18 and PAM-18Na.

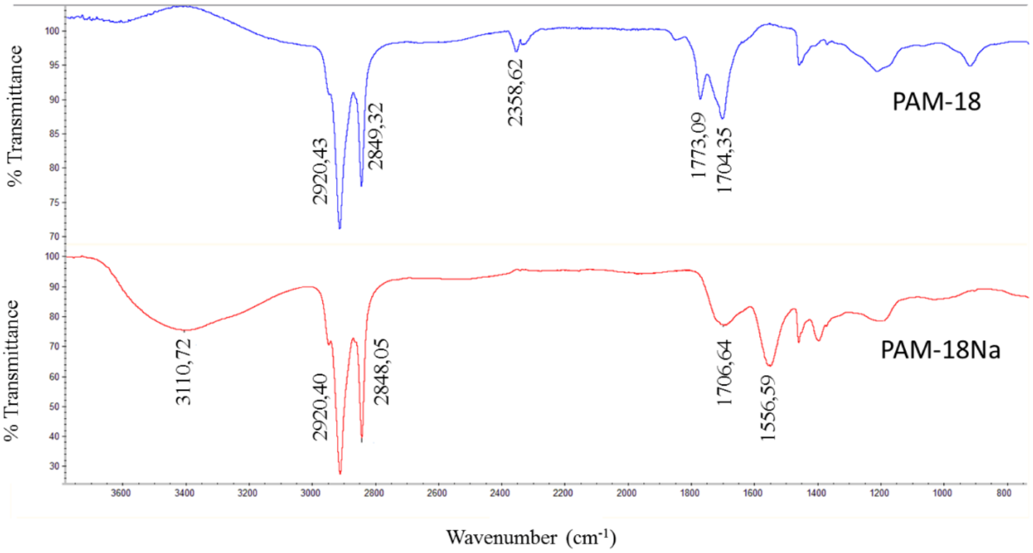

Figure. 1: FTIR of polymeric materials PAM-18 and PAM-18Na.

2. β–Lactamase production strain characterization

The *S. aureus* strains used in this study were tested for β–lactamase production using a chromogenic nitrocefin test. According to this test, strains ATCC 29213 and ATCC 43300 are β–lactamase producers. On the other hand, the strain ATCC 25293 is not. These results agree with CLSI reports for these strains and support our ampicillin disk diffusion tests. According to the literature, the strain ATCC 25293 is sensitive to ampicillin. On the other hand, the strains ATCC 29213 and ATCC 43300 are resistant to ampicillin. The resistance of the strain ATCC 29213 is attributed only to the secretion of β–lactamase enzymes to the media. However, for the strain ATCC 43300 more than one resistance mechanism exists. In this case, the resistance arises from β–lactamase secretion and penicillin-binding protein modification [37,38]. Accordingly, we observed a 40% smaller diameter halo in the disk diffusion tests for the strain showing two resistance mechanisms (ATCC 43300). Based on these results, the two ampicillin

resistant strains selected for this study are good candidates to challenge the ability of the polymer, PAM-18Na, to protect ampicillin against enzymatic inactivation in live cells.

Determination of the ampicillin minimum inhibitory concentration (MIC)

Table 1 summarizes the calculated ampicillin MIC values for the three *S. aureus* strains used in this study.

Table 1: Minimum inhibitory concentration (MIC) for various *S. aureus* strains calculated using the microdilution antimicrobial susceptibility test for the antibiotic ampicillin, the polymer PAM-18 Na, and a 1:1 mixture of the two.

**Strain**	**MIC of AT (µg/mL)**	**MIC of PAM-18Na (µg/mL)**	**MIC of PAM-18 Na-AT (µg/mL)**
S. aureus ATCC 25923	0.12	–	0.12
S. aureus ATCC 29213	8.0	–	2.0
S. aureus ATCC 43300	32	–	8.0

– No antibiotic effect, up to the maximum concentration evaluated (256 µg/mL)

As expected from the previous experiments reported here, and the M100 guide from CLSI [36] only the resistant strains ATCC 29213 and ATCC 43300 required ≥ 0,25 µg/mL AT for growth inhibition. Also, consistent with having more than one resistance mechanism the strain ATCC 43300 required for times more antibiotic for growth inhibition than the other resistant strain ATCC 29213 and two-hundred and sixty times more antibiotic than the sensitive strain ATCC 25293.

Information on disk diffusion test is attached.

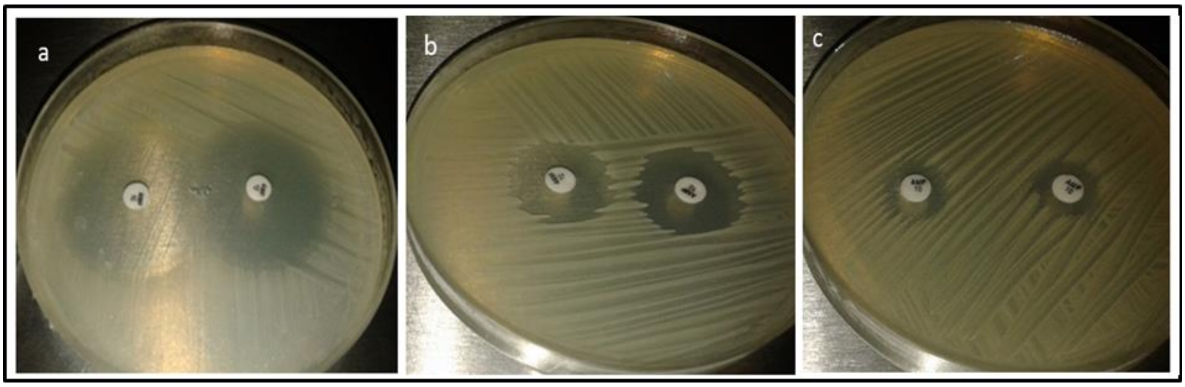

Disk diffusion test. a. *S. aureus* ATCC 25923, b. *S. aureus* ATCC 29213, c. *S. aureus* ATCC 43300 .

Information on detecting the production of enzymes β-lactamases Attached

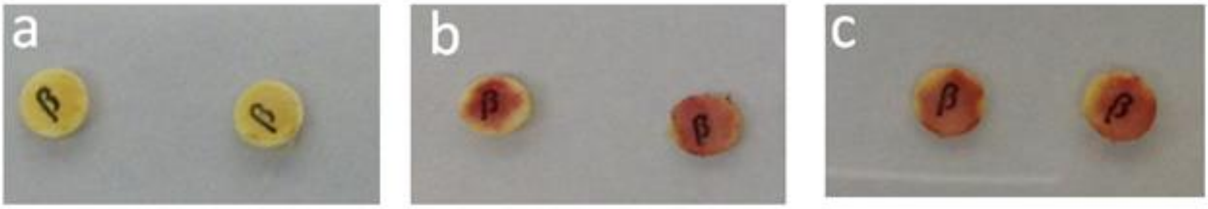

Detection of β-lactamase-producing enzymes- chromogenic nitrocefin test. A. *S. Aureus* ATCC 25923, b. *S. Aureus* ATCC 29213, c. *S. Aureus* ATCC 43300

3. Drug-polymer complex size distribution**.**

The size distribution for the drug-polymer complex is showed as raw-data obtained from the Zsizer nano ZSP instrument:

**Temperature (°C):**	25,0	**Duration Used (s):**	50
**Count Rate (kcps):**	146,7	**Measurement Position (mm):**	5,50
**Cell Description:**	Clear disposable zeta cell	**Attenuator:**	9

			**Size (d.nm):**	**% Intensity:**	**St Dev (d.nm):**
**Z-Average (d.nm):**	161,1	**Peak 1:**	221,0	82,3	102,5
**PdI:**	0,455	**Peak 2:**	44,72	11,3	12,36
**Intercept:**	0,965	**Peak 3:**	4833	6,5	714,4
**Result quality :**	Good				

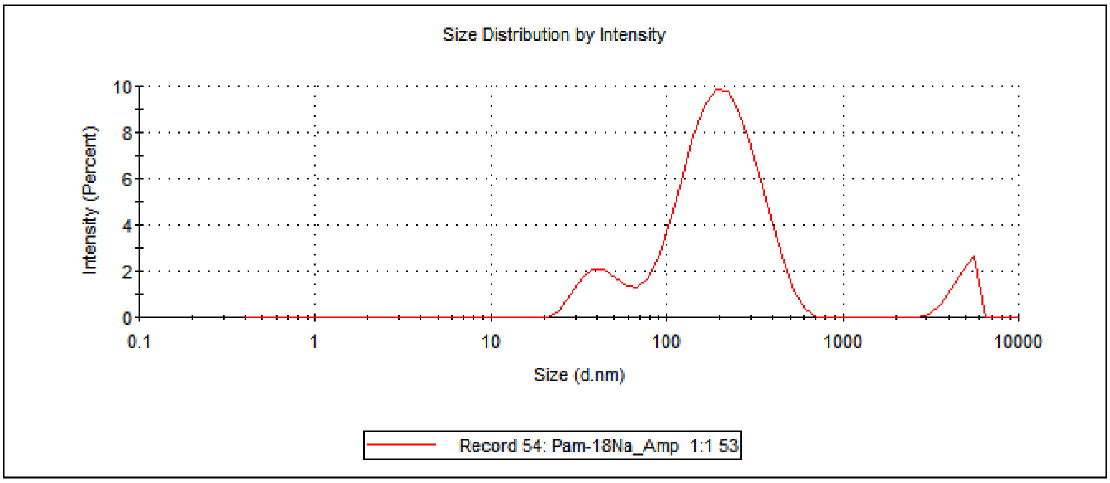

It could be observed in the figure, the presence of three size populations in the sample. The size distributions around 221.0 nm with 82.3% of intensity, 44.72 nm with 11.3% of intensity and one big population around 4833 nm with 6.5%. All of these are shifted when the pH value of the system changes, this behavior has been already reported in the manuscript.
